# Human Cytomegalovirus miR-US25-1 Targets the GTPase RhoA To Inhibit CD34^+^ Hematopoietic Progenitor Cell Proliferation To Maintain the Latent Viral Genome

**DOI:** 10.1128/mBio.00621-21

**Published:** 2021-04-06

**Authors:** Nicole L. Diggins, Lindsey B. Crawford, Meaghan H. Hancock, Jennifer Mitchell, Jay A. Nelson

**Affiliations:** a Vaccine and Gene Therapy Institute, Oregon Health and Science University, Beaverton, Oregon, USA; Princeton University

**Keywords:** CD34^+^ hematopoietic progenitor cells, human cytomegalovirus, latency, RhoA, miRNA

## Abstract

Human cytomegalovirus (HCMV) microRNAs play essential roles in latency and reactivation in CD34^+^ hematopoietic progenitor cells (HPCs) via regulation of viral and cellular gene expression. In the present study, we show that HCMV miR-US25-1 targets RhoA, a small GTPase required for CD34^+^ HPC self-renewal, proliferation, and hematopoiesis. Expression of miR-US25-1 impairs signaling through the nonmuscle myosin II light chain, which leads to a block in cytokinesis and an inhibition of proliferation. Moreover, infection with an HCMV mutant lacking miR-US25-1 resulted in increased proliferation of CD34^+^ HPCs and a decrease in the proportion of genome-containing cells at the end of latency culture. These observations provide a mechanism by which HCMV limits proliferation to maintain latent viral genomes in CD34^+^ HPCs.

## Abstract (precis)

Each herpesvirus family establishes latency in a unique cell type. Since herpesviruses genomes are maintained as episomes, the viruses need to devise mechanisms to retain the latent genome during cell division.

## INTRODUCTION

Human cytomegalovirus (HCMV), a betaherpesvirus, is a widespread pathogen, with 30% of children infected by age 5 and between 40 and 90% adults infected worldwide (1, 2). After an initial acute infection, HCMV establishes latency in CD14^+^ monocytes and CD34^+^ hematopoietic progenitor cells (HPCs), resulting in lifelong infection of the host (3–7). The latent state of infection is characterized by limited viral gene expression without infectious virus production. Periodic reactivation of the virus from latency is typically controlled by a robust T cell response (8), but reactivation can lead to uncontrolled virus replication and significant disease in immunocompromised patients (9) and remains a significant cause of morbidity and mortality after solid organ transplantation (10) and hematopoietic stem cell transplantation (11). HCMV reactivation in CD34^+^ HPCs is exquisitely linked to differentiation into myeloid lineage cells (3, 12), and thus cellular signals that stimulate differentiation of CD34^+^ HPCs along the myeloid lineage can trigger HCMV reactivation. Viral regulation of these cellular processes determines whether latency is maintained or reactivation is initiated.

Since their initial discovery in the Herpesviridae family, viral microRNAs (miRNAs) have been postulated to play important roles in viral latency and persistence. miRNAs are short, ∼22-nucleotide single-stranded RNA species that posttranscriptionally regulate gene expression by targeting the RNA-induced silencing complex (RISC) to mRNAs that have partially complementary sequences (13). miRNAs are nonimmunogenic and can target hundreds of transcripts, making these RNA species a particularly advantageous mechanism for HCMV to manipulate both viral and cellular genes during infection (14). Alpha- and gammaherpesviruses encode miRNAs in regions that are associated with latent gene expression (15, 16), and the roles of miRNAs during latency were initially uncovered for Kaposi’s sarcoma-associated herpesvirus (KSHV) and Epstein-Barr virus (EBV) (17, 18). The functions of HCMV-encoded miRNAs in latency have been more difficult to elucidate, in part due to the fact that HCMV miRNAs are scattered throughout the genome and are not known to associate with any particular latency expression profile but also because of the difficulty in utilizing appropriate model systems to study HCMV latency.

Recent evidence indicates that a subset of the 22 HCMV-encoded miRNAs are expressed during latent infection of CD34^+^ HPCs (19–21), suggesting that these viral gene products play important roles in latency establishment and maintenance, as well as in sensing signals for viral reactivation. Indeed, many targets have been identified for the HCMV miRNAs that modulate processes including viral replication (22–24), virion assembly (23), cytokine secretion (20, 23, 25–27), immune evasion (28–31), cell survival (32–37), and the cell cycle (21, 22, 38). Although much of the work on HCMV miRNAs has investigated their roles in lytic infection, their roles in regulating the cellular environment likely also extend to latent infection (14). Emerging evidence points to a role of HCMV miRNAs in controlling latency and reactivation events. miR-US22 inhibits epidermal growth factor receptor (EGFR) signaling through targeting of the transcription factor Egr-1 to promote myelopoiesis and viral reactivation (19). Transforming growth factor beta (TGF-β) production and signaling has also been implicated in HCMV latency. miR-US5-2 targets the transcriptional repressor NAB1 to promote TGF-β production and inhibit myelopoiesis of CD34^+^ HPCs. Conversely, miR-UL22A targets SMAD3 to block TGF-β signaling within the infected cell, which is required to maintain viral genomes during latency (39). miR-UL148D targets immediate early response gene 5 (IER5) (21) and the activin receptor ACVR1B (20), which have been shown to limit IE gene expression and immune detection, respectively, in models of latent infection. Though the role of HCMV miRNAs in latency and reactivation are becoming increasingly appreciated, the precise pathways regulated by HCMV miRNAs in cells that support viral latency remain to be fully elucidated.

Rho GTPases are critical regulators of the actin cytoskeleton and play many roles in cellular processes such as adhesion, proliferation, and migration (40). These GTPases are activated by guanine nucleotide exchange factors in response to various upstream receptor signals (41). Once activated, Rho family proteins activate multiple effector proteins to induce actin cytoskeleton rearrangements necessary for focal adhesion formation, movement of vesicles throughout the cell, endocytosis and exocytosis, cell division, and motility (41–45). RhoA has been implicated in regulating migration during HCMV infection (46–49); however, the role of RhoA during HCMV latency has not been explored. RhoA is a Rho family GTPase that is crucial for hematopoiesis. Studies from a conditional knockout mouse model revealed that CD34^+^ HPCs lacking RhoA expression were unable to self-renew and exhibited a severe proliferation defect, resulting in a complete hematopoietic failure (50, 51). While stem cell precursors of HPCs were largely unaffected by RhoA depletion, HPCs were unable to differentiate down the myeloid lineage (51). Due to their massive regulatory potential and involvement in hematopoiesis, targeting of Rho GTPases could be a means that the virus uses to manipulate proliferation and differentiation of CD34^+^ HPCs in order to regulate reactivation.

Herpesviruses exist in latently infected cells as an episome and need mechanisms for retention in dividing cells, where the viral genome could be lost in this process. Alphaherpesviruses latently infect nondividing neurons, whereas gammaherpesviruses tether the genome to the cellular chromosome (52, 53). In the present study, we show that latently expressed HCMV miR-US25-1 downregulates expression of RhoA to inhibit CD34^+^ HPC proliferation by blocking mitosis. Mutation of miR-US25-1 results in the loss of the HCMV genome in latently infected cells. These results reveal a novel mechanism through which HCMV is able to regulate cell division to prevent the loss of the viral genome in proliferating cells.

## RESULTS

### HCMV miR-US25-1 downregulates the expression of RhoA.

**FIG 1:**
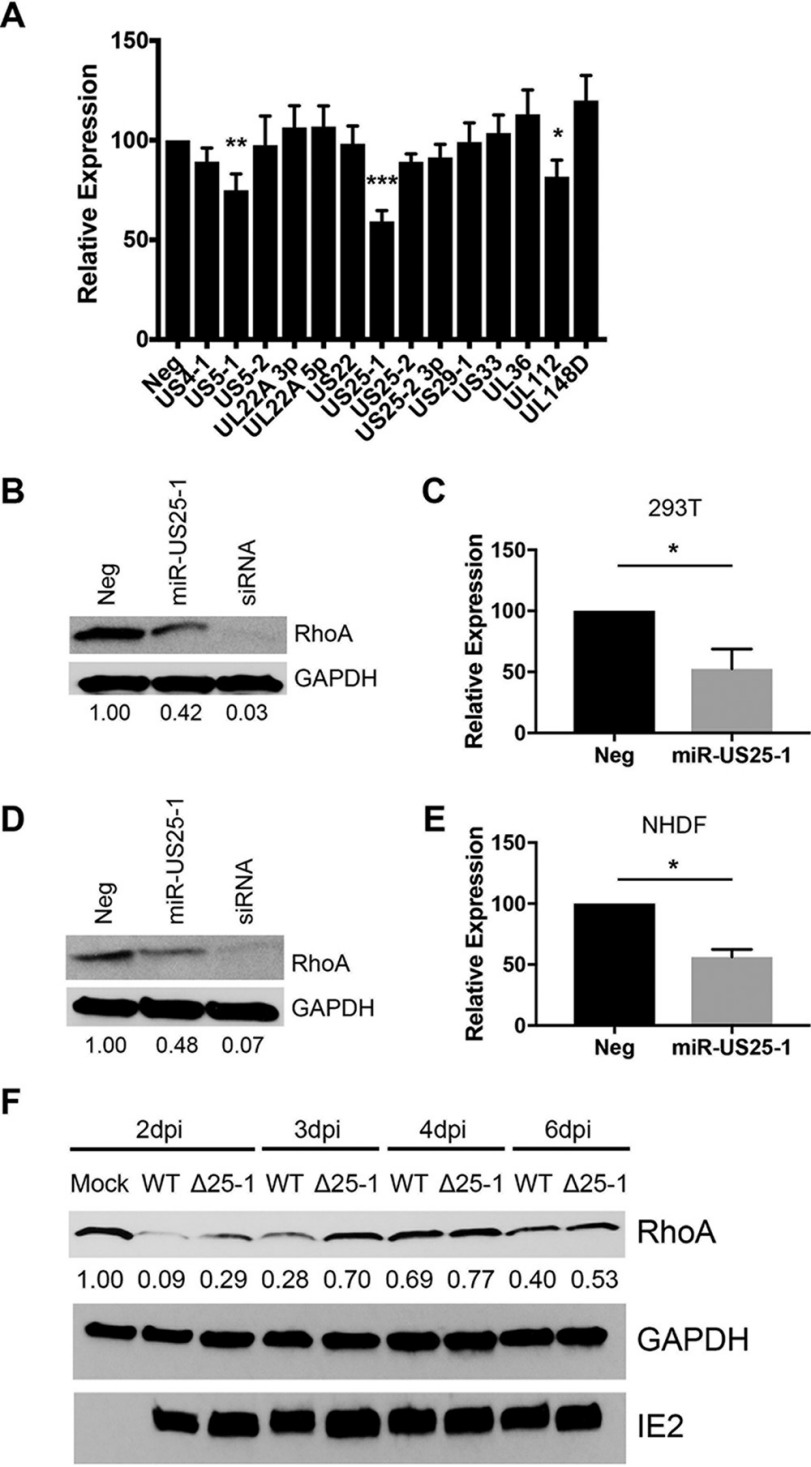
miR-US25-1 targets RhoA. (A) A dual luciferase reporter containing the 3′UTR of RhoA was cotransfected into HEK293T cells, along with double-stranded miRNA mimics or negative control (Neg). Luciferase expression was assessed after 24 h. The relative expression is shown as a percentage of Neg. Error bars represent the standard errors of the mean from four separate experiments (*, *P* < 0.05; **, *P* < 0.005 ***, *P* < 0.0001 [one-way ANOVA]). HEK293T (B) or NHDF (D) cells were transfected with miRNA mimics or siRNA. Lysates were harvested 72 h posttransfection and immunoblotted for RhoA and GAPDH (loading control). Quantification shows relative expression levels compared to Neg. (C and E) Quantification of data from panels B and D, respectively, from three independent experiments. Error bars represent standard errors of the mean (*, *P* < 0.05 [unpaired *t* test]). (F) NHDF cells were infected at an MOI of 3 PFU/cell with WT HCMV (TB40/E-GFP), a mutant lacking miR-US25-1 (Δ25-1), or uninfected (Mock). Lysates were harvested after 2, 3, 4, or 6 dpi and immunoblotted for HCMV IE2, RhoA, and GAPDH. Quantification shows the relative expression levels of RhoA compared to GAPDH and normalized to Mock.

Since RhoA has been implicated in CD34^+^ HPC self-renewal and hematopoiesis (50), we sought to determine whether RhoA is targeted by HCMV miRNAs. To this end, we transfected HCMV or negative-control miRNA mimics into HEK293T cells, along with a luciferase reporter plasmid containing the 3′ untranslated region (3′UTR) of RhoA. The expression of miR-US5-1, miR-US25-1, and miR-UL112 significantly reduced luciferase expression compared to the negative control (Fig. 1A), suggesting that RhoA is a target of these miRNAs. Expression of miR-US25-1 reduced endogenous protein levels of RhoA in both HEK293T cells and normal human dermal fibroblasts (NHDFs) by ∼50% (Fig. 1B to E). However, neither miR-US5-1 nor miR-UL112 affected RhoA expression at the protein level (see Fig. S1 in the supplemental material). To assess the ability of miR-US25-1 to downregulate RhoA expression in the context of HCMV infection, we used bacterial artificial chromosome (BAC) recombineering to generate a mutant virus lacking the miR-US25-1 hairpin in HCMV TB40E-GFP. NHDFs were infected with wild-type (WT) HCMV or ΔmiR-US25-1, and whole-cell lysates were harvested at 2, 3, 4, and 6 days postinfection (dpi). Western blot analysis demonstrated increased RhoA expression in ΔmiR-US25-1-infected cells compared to WT HCMV at all time points (Fig. 1F), suggesting that miR-US25-1 targets RhoA during HCMV infection.

10.1128/mBio.00621-21.1 FIG S1 miR-US5-1 and miR-UL112 do not target RhoA. (A) HEK293T cells were transfected with the indicated miRNA mimics or RhoA siRNA. Lysates were harvested at 72 h posttransfection and immunoblotted for RhoA and GAPDH (loading control). Quantification shows relative expression levels compared to GAPDH and normalized to Neg. (B) Quantification of panel A from three separate experiments. Error bars represent standard error of the mean from three separate experiments. *, *P* < 0.05; NS, not significant (one-way ANOVA compared to Neg). Download FIG S1, TIF file, 0.4 MB. Copyright © 2021 Diggins et al. 2021 Diggins et al. https://creativecommons.org/licenses/by/4.0/ This content is distributed under the terms of the Creative Commons Attribution 4.0 International license.

**FIG 2:**
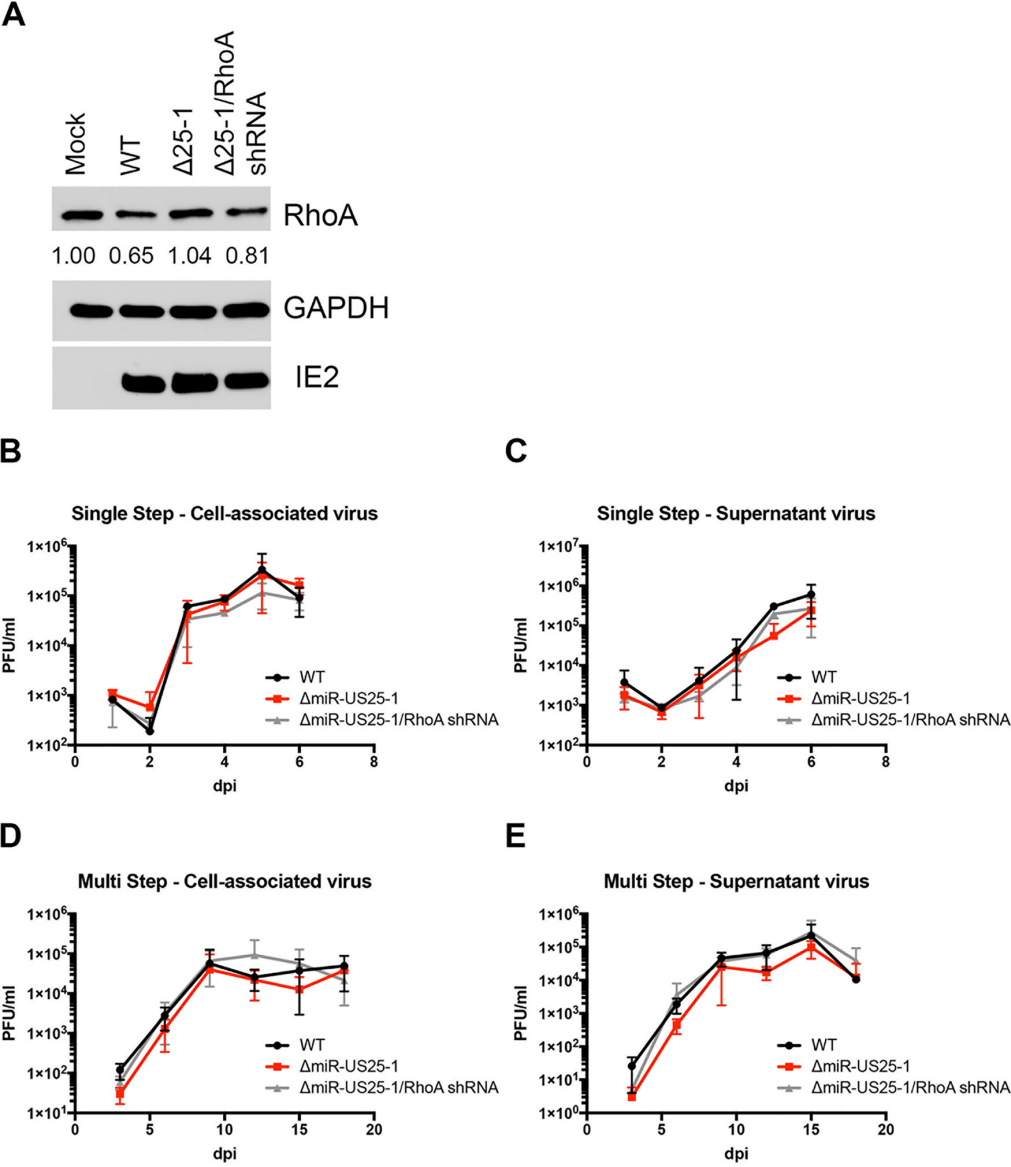
Characterization of miR-US25-1 mutant viruses. (A) NHDFs were infected at an MOI of 3 with WT HCMV TB40E, a mutant lacking miR-US25-1 (Δ25-1), or a mutant expressing a RhoA shRNA in the place of miR-US25-1 (Δ25-1/RhoA shRNA). Lysates were harvested after 72 h and immunoblotted for HCMV IE2, RhoA, and GAPDH. Quantification shows the relative expression levels of RhoA compared to GAPDH and normalized to Mock. (B to E) NHDF cells were infected with WT HCMV or miR-US25-1 mutants at an MOI of 3 for single-step (B and C) or an MOI of 0.01 for multistep (D and E) growth curves. The PFU/ml values were quantified in duplicate from samples collected at the indicated time points for cell-associated (B and D) or supernatant (C and E) virus.

Since an individual miRNA can potentially target hundreds of genes (54), we wanted to assess the role of miR-US25-1 specifically targeting RhoA during HCMV infection. To this end, we generated a recombinant virus where miR-US25-1 is replaced with a RhoA shRNA (ΔmiR-US25-1/RhoA shRNA) using BAC recombineering. NHDFs were infected with HCMV WT, ΔmiR-US25-1, or ΔmiR-US25-1/RhoA shRNA for 3 days, and then whole-cell lysates were harvested and immunoblotted for RhoA. Although WT HCMV-infected cells demonstrated decreased RhoA expression compared to mock-infected cells, ΔmiR-US25-1 infection resulted in increased RhoA expression compared to WT HCMV-infected fibroblasts and expression similar to that of mock-infected cells (Fig. 2A). In addition, expression of a RhoA shRNA in the context of a miR-US25-1 deletion reduced RhoA levels compared to cells infected with the ΔmiR-US25-1 mutant virus. Both ΔmiR-US25-1 and ΔmiR-US25-1/RhoA shRNA viruses grew with WT kinetics (Fig. 2B to E), suggesting that the effects on RhoA expression are not due to changes in the replication of the mutant viruses. Taken together, the data indicate that RhoA is a miR-US25-1 target during HCMV infection.

**FIG 3:**
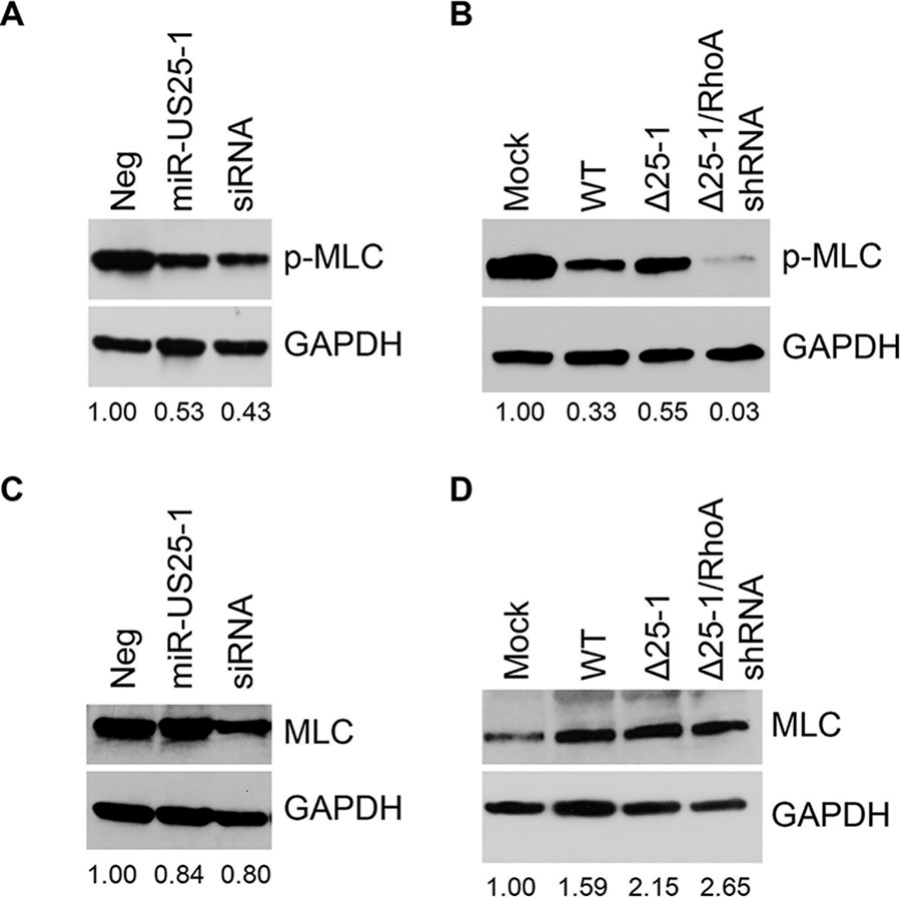
miR-US25-1 impairs RhoA signaling through myosin II. NDHF cells were transfected with miRNA mimics or RhoA siRNA (A and C) or infected with WT HCMV or miRNA mutants at an MOI of 3 (B and D). Lysates were harvested after 72 h and immunoblotted for p-MLC (A and B) or total MLC (C and D), with GAPDH as a loading control. Quantification shows the relative expression levels of p-MLC (A and B) or MLC (C and D) normalized to Neg (A and C) or Mock (B and D).

We next sought to determine whether expression of miR-US25-1 has an effect on downstream RhoA signaling. Active RhoA binds to and regulates the activity of multiple effector proteins, including Rho kinase (ROCK), myosin light chain kinase (MLCK), mDia1, and LIM kinase (LIMK), which together promote actomyosin contractility through phosphorylation of the myosin light chain (MLC) of nonmuscle myosin II at Ser19 (55). We therefore tested the ability of miR-US25-1 to alter MLC phosphorylation. Ectopic expression of miR-US25-1 decreased levels of MLC phosphorylation in NHDF cells by ∼50%, comparable to knockdown of RhoA by siRNA (Fig. 3A, S2A). In agreement with this, p-MLC levels were reduced ∼70% during infection with WT HCMV (Fig. 3B; see also Fig. S2B in the supplemental material). Infection with ΔmiR-US25-1, however, increased p-MLC levels compared to WT HCMV-infected cells. Expression of a RhoA shRNA in place of miR-US25-1 was able to reduce p-MLC levels, suggesting that the virus-mediated effects on p-MLC are at least partially dependent on miR-US25-1 regulation of RhoA expression. Total MLC levels were slightly decreased by miR-US25-1 (Fig. 3C; see also Fig. S2C) similar to expressing a RhoA siRNA, indicating that the effect of miR-US25-1 is predominantly on activation of MLC and not total protein levels. Interestingly, while HCMV infection decreased MLC phosphorylation (Fig. 3B; see also Fig. S2B), total MLC expression was induced by the virus (Fig. 3D; see also Fig. S2D). This induction of MLC expression by HCMV is not driven by miR-US25-1, since increased total MLC levels were also observed with infection with ΔmiR-US25-1 and ΔmiR-US25-1/RhoA shRNA viruses. This observation suggests that despite the increased amount of MLC present during HCMV infection, phosphorylation of MLC is significantly impaired during infection by miR-US25-1 targeting of RhoA.

10.1128/mBio.00621-21.2 FIG S2 miR-US25-1 impairs RhoA signaling through myosin II. Additional Western blots related to Fig. 3 are shown. NDHF cells were transfected with miRNA mimics or RhoA siRNA (A and C) or infected with WT HCMV or miRNA mutants at an MOI of 3 (B and D). Lysates were harvested after 72 h and immunoblotted for p-MLC (A and B) or total MLC (C and D), with GAPDH as a loading control. Quantification shows the relative expression levels of p-MLC (A and B) or MLC (C and D) normalized to Neg (A and C) or Mock (B and D). Download FIG S2, TIF file, 0.5 MB. Copyright © 2021 Diggins et al. 2021 Diggins et al. https://creativecommons.org/licenses/by/4.0/ This content is distributed under the terms of the Creative Commons Attribution 4.0 International license.

### HCMV miR-US25-1 inhibits the proliferation of fibroblasts by blocking cytokinesis.

**FIG 4:**
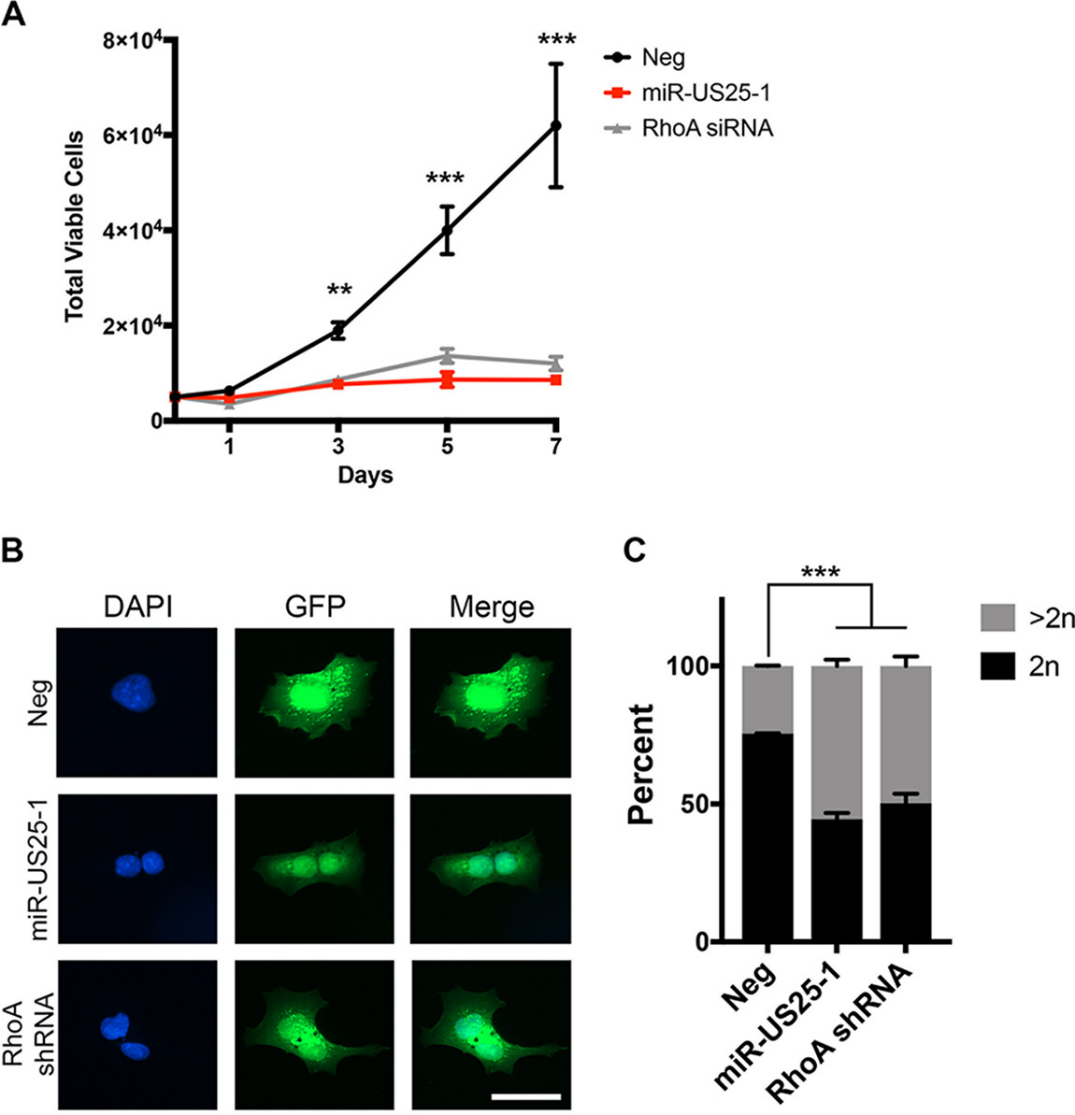
miR-US25-1 inhibits proliferation of fibroblasts. (A) NDHF cells were transfected with miRNA mimics or siRNA. After 72 h, 5 × 10^3^ cells were plated in duplicate in 12-well tissue culture dishes and total live cells were determined by trypan blue exclusion counting at 1, 3, 5, and 7 days. Error bars represent standard errors of the mean from three separate experiments (**, *P* < 0.005; ***, *P* < 0.001 [one-way ANOVA, compared to Neg]). (B) HEK293 cells were transfected with pSIREN plasmids expressing miR-US25-1, RhoA shRNA, or empty vector (Neg). At 24 h posttransfection, the cells were plated onto coverslips, fixed 48 h later, and stained for DAPI. Scale bar, 50 μm. (C) Quantification from panel B. The numbers of nuclei were quantified for each GFP-positive cell. Stacked bars represent percentage of cells containing either one nucleus (2n) or two or more nuclei (>2n). At least 200 cells were analyzed per condition. Error bars represent standard errors of the mean from three separate experiments (***, *P* < 0.001 [one-way ANOVA, compared to Neg]).

**FIG 5:**
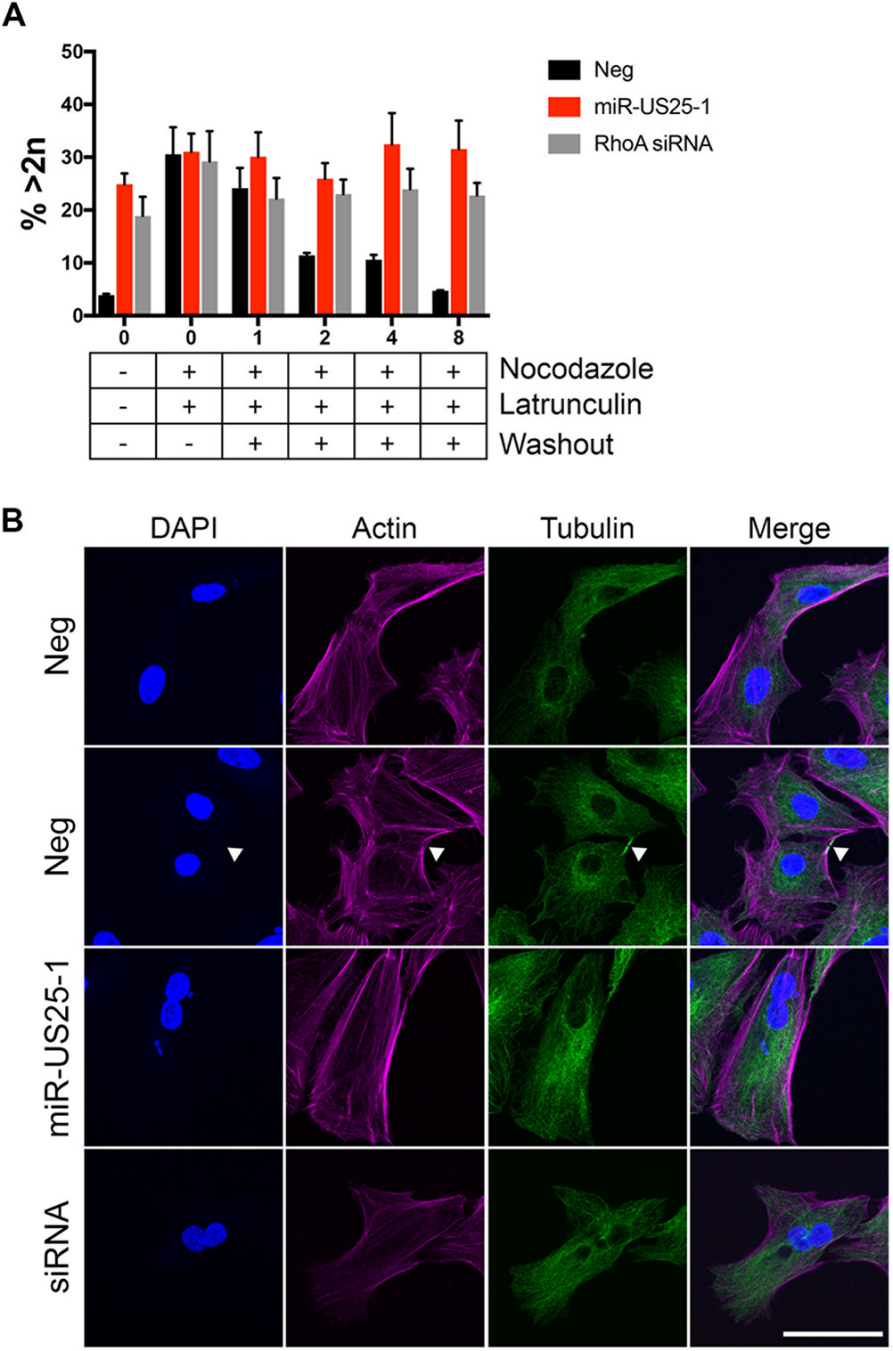
RhoA downregulation by miR-US25-1 disrupts cytokinesis. NHDF cells were plated on coverslips and transfected with miR-US25-1, RhoA siRNA, or negative control miRNA mimic. At 48 h posttransfection, the cells were treated with 1 μg/ml nocodazole for 20 h, followed by 0.1 μg/ml latrunculin for 30 min. Nocodazole and latrunculin were washed out with fresh media, and the cells were incubated for 0 to 8 h before fixation. The cells were stained for actin (phalloidin), tubulin, and nuclei (DAPI). (A) The number of nuclei were quantified for each cell. At least 700 cells were counted per group from three separate experiments. The graph shows the percentages of cells that contained more than one nucleus (>2n) at each time point. (B) Representative images from panel A. White arrowheads indicate midbody formation. Scale bar, 50 μm.

RhoA-mediated activation of myosin II promotes actomyosin contractility, which is important for a variety of cellular functions, including proliferation (44, 56, 57). Nonmuscle myosin II cross-links actin filaments to form the actin bundles required for contractile ring formation during cytokinesis (58). Since other HCMV miRNAs have been shown to affect cellular proliferation (19, 22, 24, 39), and miR-US25-1 reduces RhoA signaling through myosin II, we hypothesized that miR-US25-1 would also alter proliferation. We performed proliferation assays with NHDFs transfected with a miR-US25-1 mimic, RhoA siRNA, or a negative control. Both miR-US25-1 and RhoA knockdown decreased proliferation of fibroblasts by 7-fold over the course of 7 days (Fig. 4A). This effect on proliferation was not due to an increase in cell death, since cell viability was not affected by miR-US25-1 expression or RhoA knockdown (see Fig. S3). We hypothesized that miR-US25-1 would affect proliferation through regulation of cytokinesis, since RhoA signaling and actomyosin contractility is required for the completion of cell division (58). When miR-US25-1 or RhoA shRNA were ectopically expressed in HEK293 cells, we observed a 2-fold increase in the presence of binucleate and multinucleate cells (Fig. 4B and C), a scenario that occurs when cells go through mitosis and yet are unable to fully divide during cytokinesis (59, 60). To observe the effect of miR-US25-1 on cytokinesis directly, NHDFs expressing miR-US25-1, RhoA siRNA, or the negative control were treated with nocodazole for 20 h to prevent microtubule assembly and thereby enrich for cells in mitosis. After nocodazole treatment, the cells were treated with the actin polymerization inhibitor latrunculin, which allows cells to proceed to late mitosis but prevents cytokinesis. Nocodazole and latrunculin were then washed out to allow cells to complete mitosis. In negative control-transfected cells, treatment with nocodazole and latrunculin increased the number of binucleate cells from 3.8 to 31% (Fig. 5A). Following washout, the majority of control cells were able to complete cytokinesis; by 8 h after washout, the number of binucleate cells had returned to a similar level as prior to cytoskeletal drug treatment (Fig. 5A). Nocodazole and latrunculin treatment of miR-US25-1 or RhoA siRNA-transfected cells also increased the number of binucleated cells to 30 and 29%, respectively, although these cells were already enriched for binucleated cells prior to nocodazole and latrunculin treatment (24 and 19%, respectively). A similar number of cells remained binucleated 8 h after washout compared to miR-US25-1 or RhoA-expressing cells prior to nocodazole and latrunculin treatment (Fig. 5A), and the total number of binucleated cells remained higher than that for control treated cells, suggesting that these cells were unable to complete cell division. Microtubule and actin staining at 8 h after washout visually confirmed that negative control-transfected cells did not show a defect in cell division, since cells either contained one nucleus (Fig. 5B, top panel) or were at the end of cytokinesis, as identified by the presence of microtubule midbodies (Fig. 5B, second panel, arrow). However, cells expressing miR-US25-1 or RhoA siRNA were not observed in cytokinesis, as evidenced by the absence of midbodies. Actin and microtubule staining showed that a subset of cells expressing miR-US25-1 or RhoA siRNA (32 and 23%, respectively; Fig. 5A) contained two or more nuclei (Fig. 5B, bottom panels), suggesting that these cells were unable to complete cytokinesis. These data imply that one mechanism whereby miR-US25-1 decreases proliferation is by impeding cytokinesis.

10.1128/mBio.00621-21.3 FIG S3 miR-US25-1 does not affect cell viability. NDHF cells were transfected with miRNA mimics or siRNA. After 72 h, 5 × 10^3^ cells were plated in duplicate in 12-well tissue culture dishes, and live and dead cells were counted at 1, 3, 5, and 7 days. The percent viability is shown as the percentages of live cells compared to total cells counted. Error bars represent the standard deviations from two separate experiments. NS, not significant (two-way ANOVA). Download FIG S3, TIF file, 0.3 MB. Copyright © 2021 Diggins et al. 2021 Diggins et al. https://creativecommons.org/licenses/by/4.0/ This content is distributed under the terms of the Creative Commons Attribution 4.0 International license.

### HCMV miR-US25-1 reduces CD34^+^ HPC proliferation to prevent viral genome loss.

**FIG 6:**
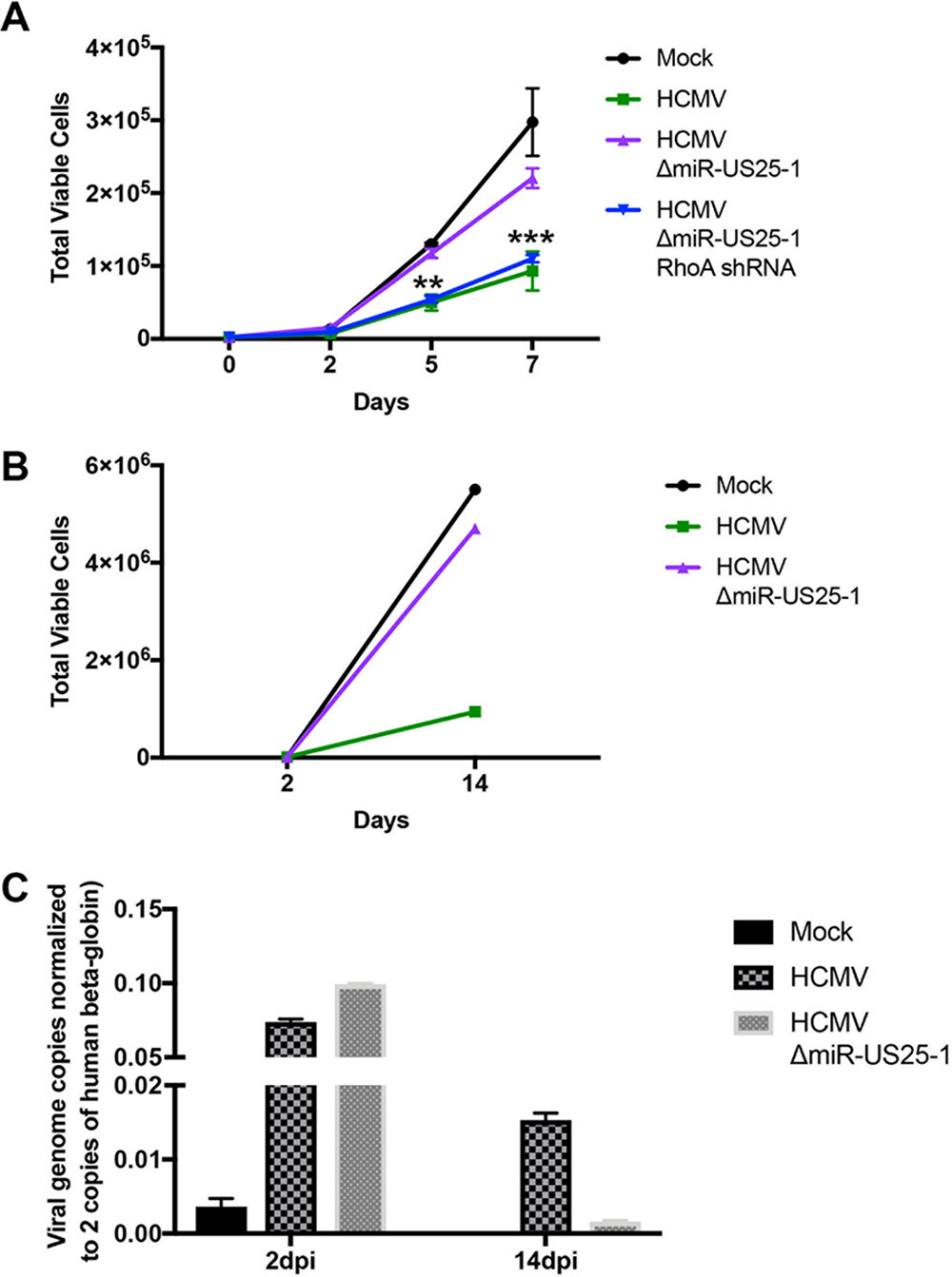
CD34^+^ HPC proliferation is impaired by miR-US25-1. CD34^+^ HPCs were infected with WT HCMV, ΔmiR-US25-1, or ΔmiR-US25-1/RhoA shRNA for 48 h. Viable CD34^+^ GFP^+^ HPCs were isolated by FACS, and uninfected cells (Mock) were isolated by FACS for viable CD34^+^ HPCs. (A) Isolated HPCs were plated in triplicate in SFEMII supplemented with hematopoietic cytokines, and total live cells were determined by trypan blue exclusion counting at day 2, 5, and 7. Error bars represent the standard deviations of triplicate wells from one representative experiment (**, *P* < 0.01; ***, *P* < 0.0001 [two-way ANOVA compared to Mock; *P* < 0.05 and *P* < 0.001, respectively, compared to ΔmiR-US25-1]). (B) Isolated HPCs were plated at equivalent numbers on stromal cell support to establish viral latency and counted after 12 days (14 dpi). The total viable cell numbers, determined by trypan blue exclusion counting, are shown from one representative experiment. (C) HCMV genome copy number in initially sorted (2 dpi) and latently infected HPCs (14 dpi) were assessed by qPCR using copies of HCMV *UL141* per two copies of human *β-globin*. Error bars represent the standard deviations of three triplicate wells from one representative experiment.

miR-US25-1 is highly expressed during latent infection (19), and so we hypothesized that the effect of this miRNA on proliferation would also occur during infection of HPCs. To test this, CD34^+^ HPCs were infected with WT TB40E-GFP, ΔmiR-US25-1, or ΔmiR-US25-1/RhoA shRNA for 48 h and then sorted for viable, CD34^+^, GFP^+^ infected cells. Isolated HPCs were plated in cytokine-rich media to promote proliferation, and the total cell numbers were counted at 2, 5, and 7 days postplating. WT HCMV-infected HPCs showed decreased proliferation compared to mock-treated cells, as has been previously observed (19, 39). ΔmiR-US25-1-infected HPCs, however, showed a 2-fold increase in proliferation compared to WT HCMV (Fig. 6A). HPCs infected with a virus expressing a RhoA shRNA in place of miR-US25-1 phenocopied the proliferation rate of WT HCMV-infected HPCs, suggesting that the effect of miR-US25-1 on HPC proliferation is dependent on RhoA targeting. We also tested the role of miR-US25-1 on proliferation in latent HCMV infection, where infected HPCs were plated on stromal cell support for 12 days in order to establish latency. Equal numbers of cells were plated at the beginning of latency, and total cells were counted at the end of the experiment. A similar trend was observed of decreased proliferation in WT HCMV-infected HPCs but not in HPCs infected with ΔmiR-US25-1 (Fig. 6B) compared to mock-infected cells. To assess whether this increase in proliferation by HCMV ΔmiR-US25-1 affected the number of cells containing viral genomes, HCMV genome copies/cell were quantified in CD34^+^ HPCs at 2 and 14 dpi. CD34^+^ HPCs latently infected with ΔmiR-US25-1 contained fewer genomes compared to WT at 14 dpi (Fig. 6C), suggesting that miR-US25-1 promotes the retention of viral genomes during latency. Collectively, these data indicate that miR-US25-1 regulation of RhoA to limit CD34^+^ HPC proliferation prevents the loss of viral genome-containing cells during latency.

## DISCUSSION

**FIG 7:**
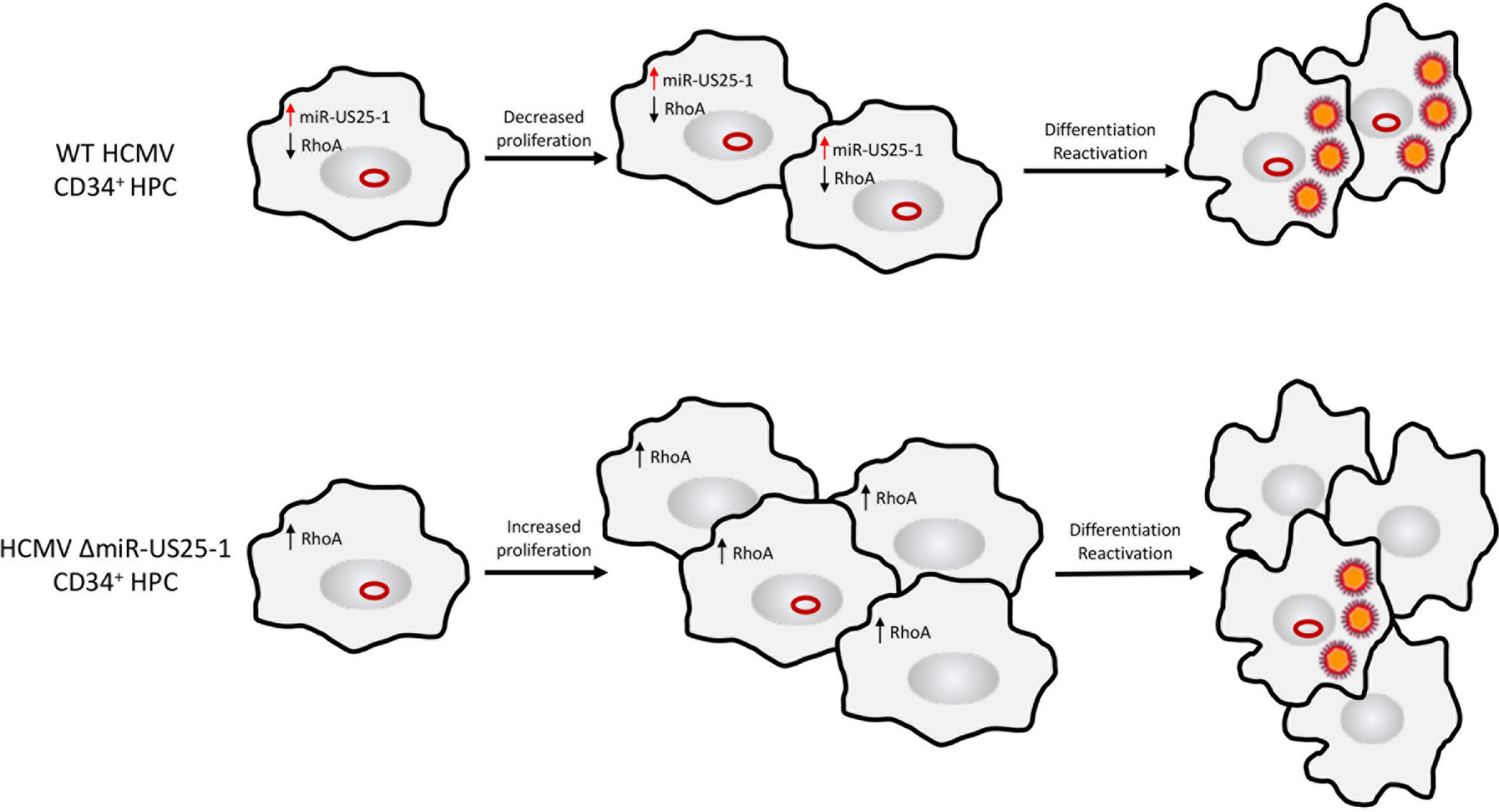
HCMV miR-US25-1-mediated regulation of RhoA expression and proliferation during latent infection of CD34^+^ HPCs. Latent infection of CD34^+^ HPCs results in the expression of HCMV miR-US25-1, which targets the GTPase RhoA to prevent proliferation and may be important for genome maintenance. When CD34^+^ HPCs are infected with a HCMV mutant that does not express miR-US25-1 (HCMV ΔmiR-US25-1), RhoA is not inhibited and increased proliferation is observed.

In the present study, we demonstrate that the GTPase RhoA is targeted for downregulation by HCMV miR-US25-1. By reducing the expression of RhoA, miR-US25-1 prevents the phosphorylation of MLC, a protein critical for bundling actin filaments. The disruption of actin filaments in turn prevents formation of the contractile ring during cytokinesis, affecting the ability of the cell to efficiently complete mitosis and proliferate. CD34^+^ HPCs infected with ΔmiR-US25-1 showed increased proliferation compared to WT HCMV-infected HPCs. Associated with the increased proliferation of ΔmiR-US25-1 latently infected cells was the loss of viral genomes. These data suggest that miR-US25-1 downregulation of RhoA is an important mechanism for retention of the viral genome in cells that can be lost through CD34^+^ HPC proliferation (Fig. 7).

Maintenance of the viral genome is critical for herpesvirus latency and lifelong infection of the host. Alphaherpesviruses maintain the viral genome through latent infection of terminally differentiated neurons that do not undergo cellular division (61). However, both betaherpesviruses and gammaherpesviruses latently infect cell with proliferative potential, presenting the problem of maintaining the viral genome without active viral replication in actively dividing cells. It is well established that gammaherpesviruses tether to host chromosomes in B cells to allow for coordinated replication of the cellular and viral genomes (62, 63). KSHV latency-associated nuclear antigen 1 (LANA-1) is responsible for binding the latent origin of replication of the KSHV genome and host cellular histones (64). EBV also has a mechanism to tether the viral genome via EBV nuclear antigen 1 (EBNA1) (52). Tethering of the viral genome to host chromosomes in gammaherpesviruses provides a mechanism to ensure that viral genomic DNA is coordinately replicated with the host cell chromosomes during mitosis. This process ensures that the viral genome is maintained in daughter cells and not lost during mitosis. Since HCMV establishes latency in CD34^+^ HPCs that proliferate to maintain progenitor cell homeostasis in the bone marrow, the virus needs a mechanism to maintain the viral genome in the cell. Some investigators have speculated that HCMV is similar to the gammaherpesviruses in tethering the viral genome to chromosomes. One study showed that a small isoform of HCMV IE1, IE1x4, binds the terminal repeat (TR) region of the HCMV genome, as well as host DNA-binding proteins, and is predicted to promote genome replication during latency (65). However, *in vitro* studies have shown that the frequency of the HCMV genome decreases during long-term culture as cells proliferate (7, 39, 66, 67). Indeed, very few cells are latently infected *in vivo* (68), suggesting that genome replication is not a major contributor to genome maintenance in latent infection. Here, we demonstrate that one potential mechanism for HCMV genome maintenance is to limit proliferation of latently infected cells. Proliferation of latently infected cells without viral genome tethering would increase the proportion of cells not carrying viral genomes while also increasing the chance of stochastic genome loss during cell division. Our data show that WT HCMV decreases proliferation of CD34^+^ HPCs compared to mock-infected cells (Fig. 6A and B) and that cells infected HCMV ΔmiR-US25-1 proliferate ∼2-fold more than the WT. Analysis of ΔmiR-US25-1 latently infected CD34^+^ HPCs for retention of viral genomic DNA after 12 days of latency revealed a significant decrease in viral genomes compared to WT HCMV (Fig. 6C). Given its effect on the proliferation of HPCs, the expression of miR-US25-1 may enrich for genome-containing cells in the CD34^+^ HPC population during *in vitro* latency assays. Since an individual miRNA can have a multitude of functions, it remains to be seen whether miR-US25-1 promotes genome retention solely through its effect on proliferation, or if an as-yet-unidentified mechanism and/or target could contribute to this process. Moreover, mutation of miR-US25-1 is not able to fully restore proliferation of HCMV-infected HPCs to mock-infected cell levels, suggesting that other viral factors are also involved in regulating proliferation of HCMV-infected HPCs. In fact, two other HCMV miRNAs, miR-US22 and miR-US5-2, contribute to HCMV-mediated effects on HPC proliferation through the modulation of EGFR and TGF-β signaling, respectively (19, 39). Evidence suggests that HCMV utilizes multiple miRNAs to effectively target broad cellular pathways by regulating expression of multiple genes within that pathway (23). Moreover, the HCMV homologue of interleukin-10 (cmvIL-10) has been shown to inhibit the proliferation of peripheral blood mononuclear cells (69). Further study is needed to understand the coordination of multiple miRNAs and/or viral proteins required for the HCMV-mediated effects on proliferation in CD34^+^ HPCs.

RhoA is a regulator of actin cytoskeletal rearrangements since GTP-loaded RhoA promotes the bundling of actin fibers through activation of nonmuscle myosin II (55, 57), which is necessary for formation of the cleavage furrow during cytokinesis (58). We show that the expression of miR-US25-1 or a RhoA siRNA decreases MLC phosphorylation (Fig. 3A). Interestingly, HCMV induces total expression of MLC compared to mock infection (Fig. 3D), and yet infected cells exhibit decreased p-MLC levels (Fig. 3B), highlighting the extent of HCMV dysregulation of myosin activation. Although WT and ΔmiR-US25-1 viruses increased total MLC levels (Fig. 3D), only ΔmiR-US25-1 increases p-MLC (Fig. 3B), suggesting that miR-US25-1 specifically acts on MLC phosphorylation. However, infection with a miR-US25-1 mutant only partially restores p-MLC levels (Fig. 3B), suggesting that other viral factors also target this signaling pathway. This could explain why proliferation is not fully restored to mock levels during infection of CD34^+^ HPCs with ΔmiR-US25-1 (Fig. 6). Other studies have demonstrated that RhoA depletion results in cells that can progress through mitosis but cannot fully divide, leading to the formation of binucleate cells (56), which supports the observations reported here (Fig. 4 and 5). However, it is unlikely that HCMV-infected cells remain binucleated. A prolonged block in mitosis can result in mitotic “slippage,” where cells undergoing prolonged cytoskeletal changes that prevent division are able to complete cell division (70, 71) but are then arrested in the G_1_ phase of the cell cycle. Thus, it is possible that proliferation is hindered not in the first round of cell division postinfection, but rather the second round of cell division would be prevented following the aberrant cell division induced by miR-US25-1 targeting of RhoA.

Studies of lytic infection models have implicated RhoA in multiple aspects of the HCMV life cycle, dependent on the cell type and stimulus used to activate the RhoA signaling pathway. Initial studies of HCMV US28 revealed that US28 signaling through Gα12 stimulates RhoA activation and subsequent migration of smooth muscle cells in response to the CC-chemokine RANTES (47, 48). In agreement with these findings, migration of HCMV-infected glioblastoma cells was impaired in cells depleted of RhoA (as well as RhoB and RhoC) (46). However, this effect appears to be both cell type and ligand specific, since stimulation of US28 by Fractalkine induced macrophage migration but inhibited RANTES-mediated smooth muscle cell migration (49). The activation of RhoA in macrophages in response to Fractalkine remains to be tested. In addition to its role in migration, RhoA has also been implicated in early steps of HCMV lytic infection, as well as immune signaling during late infection. HCMV interaction with EGFR and αVβ3 integrin receptors trigger viral entry and the rapid downregulation of RhoA and the RhoA effector cofilin. This downregulation of the RhoA-cofilin signaling axis is accompanied by the loss of stress fibers to allow for viral translocation to the nucleus (72), which occurs prior to miR-US25-1 expression. A recent study pointed to a role for RhoA in immune signaling during HCMV infection. Infection of fibroblasts induced IL-11 secretion, which increased after RhoA depletion (73) and may play a role in host cell survival. Collectively, these data suggest that RhoA activity is important for multiple parts of the HCMV life cycle, depending on the cell type infected and the signal transduction pathways induced by the virus. Our data show that loss of miR-US25-1 in HCMV did not affect viral replication in fibroblasts (Fig. 2), suggesting that miR-US25-1 is dispensable for lytic infection and may instead function during latency. Indeed, latently infected HPCs lacking miR-US25-1 have increased proliferation compared to WT-infected HPCs (Fig. 6B). Interestingly, miR-US25-1 also targets several cell cycle genes within 5′UTRs, including cyclin E2 (38). Using a reporter plasmid in which we cloned the 3′ UTR of RhoA downstream of the *Renilla* luciferase gene, we show that miR-US25-1 is able to inhibit luciferase expression (Fig. 1A). This suggests that the 3′UTR is sufficient for miR-US25-1 targeting, which is consistent with previously published data demonstrating that 3′UTR seed sequences account for ∼15% of miR-US25-1 target sites (38). It is possible that these cell cycle targets, as well as other undiscovered targets of miR-US25-1, contribute to HCMV-mediated regulation of proliferation. Nonetheless, RhoA targeting is likely the major contributor to miR-US25-1 effects on proliferation, since RhoA knockdown in the context of a miR-US25-1 mutant virus was able phenocopy WT HCMV in proliferation assays (Fig. 6). To our knowledge, our results are the first to implicate RhoA in the regulation of biological functions of CD34^+^ HPCs during HCMV latency, which may be one of the reasons that miR-US25-1 is one of the most highly expressed viral miRNAs during latency (19).

Apart from its role in proliferation, RhoA is involved in multiple signaling pathways and cellular processes that are manipulated by HCMV and as such miR-US25-1 may act cooperatively with other viral gene products to regulate these pathways. For instance, RhoA promotes actin polymerization around vesicles to contribute to vesicular trafficking (43). Since the Rho GTPase Cdc42 is targeted by viral miRNAs, along with other trafficking proteins, to allow for reorganization of the endocytic compartment to form the virion assembly compartment (VAC) (23), targeting of RhoA may also be tied to formation of the VAC. In addition, RhoA promotes canonical and noncanonical NF-κB signaling (74), which are highly regulated by HCMV (75). miR-US5-1 and miR-UL112-3p inhibit NF-κB signaling by targeting IKKα and IKKβ (28). miR-US25-1, through downregulating RhoA, may also interfere with NF-κB signaling and cytokine production. Recent evidence from CD34^+^ HPCs demonstrated that the TGF-β signaling is blocked by HCMV to maintain latency through miR-UL22A-mediated targeting of SMAD3 (39). The RhoA signaling pathway can be activated by noncanonical TGF-β signaling in a cell-specific manner (76, 77), so the downregulation of RhoA by miR-US25-1 may represent another means by which HCMV modulates TGF-β signaling. RhoA is also downstream of EGFR (78), which is a central regulator of the HCMV latency program (67, 79, 80). EGFR signaling is required for the establishment of latency, while attenuation of EGFR signaling is thought to trigger reactivation of HCMV in CD34^+^ HPCs. EGFR signaling is tightly regulated by several viral factors, including UL135, UL138, and miR-US22 (19, 79–84). Evidence from lytic infection studies suggests that RhoA is downregulated upon HCMV interaction with EGFR at the cell surface (72); however, the role of RhoA in EGFR signaling during latency has not yet been demonstrated. Further study will be required to unravel the complex, interconnected cell signaling pathways regulated by miR-US25-1.

HCMV infects multiple cell types and has a broad tissue tropism (85, 86). For each cell type, the virus exhibits unique methods of manipulating the cellular environment to achieve discrete modes of infection. In CD34^+^ HPCs, HCMV manipulates cellular processes that are important for establishing and maintaining latency or triggering reactivation events. Rho GTPase signaling is a central player in a vast array of biological functions and represents an attractive means for HCMV to influence the host cell. Our data suggest that HCMV miR-US25-1 is important for limiting proliferation of infected CD34^+^ HPCs during latent infection in order to enrich for genome-containing cells. Additional studies on how this miRNA interacts with other viral factors expressed during latency will be required to gain a more comprehensive understanding of HCMV latency.

## MATERIALS AND METHODS

### Cells and media.

HEK293 and adult normal human dermal fibroblasts (NHDFs) were obtained from the American Type Culture Collection (ATCC) and cultured in Dulbecco modified Eagle medium (DMEM) supplemented with 10% heat-inactivated fetal bovine serum (FBS; HyClone), 100 U/ml penicillin, 100 μg/ml streptomycin, and 100 μg/ml glutamine (Thermo Fisher). M2-10B4 and S1/S1 stromal cells were obtained from Stem Cell Technologies and maintained in DMEM with 10% FBS and penicillin, streptomycin, and glutamine as previously described (87). CD34^+^ hematopoietic progenitor cells (HPCs) were isolated from deidentified human fetal liver obtained from Advanced Bioscience Resources as previously described (88). All cells were maintained at 37°C and 5% CO_2_.

### Viruses.

Viruses used in this study include BAC-generated WT TB40/E expressing GFP from the SV40 promoter (89, 90), and TB40/E mutant viruses lacking the pre-miR-US25-1 sequence or with a RhoA shRNA replacing miR-US25-1 were generated by galactokinase (GalK)-mediated recombination (91). Briefly, the *galK* gene was used to replace the miR-US25-1 pre-miRNA hairpin using homologous recombination (miR-US25-1 galK F, CACCGTCACCCCGCTCCCAAGCGCCGCGAAAAGTGCTCCGATTTTTCACCGTCGTTCGCGACGTTGATTTGCCTCGCCTGTTGACAATTAATCATCGGCA; miR-US25-1 galK R, GCGGGCGCGGGGTGGCGAAGCGGGGAGCGCCGATGTACCTGCAGCTCGAACGTCTCTCCGGTAACTATCGGCGGCCGGGGCTCAGCAAAAGTTCGATTTA). In the second recombination step, *galK* is removed using oligonucleotides that encompass the regions up- and downstream of the pre-miR-US25-1 sequence (miR-US25-1 F, GCGACGTTGATTTGCCTCGGTCGCCCCGGCCGCCGATAGTTA; miR-US25-1 R, TAACTATCGGCGGCCGGGGCGACCGAGGCAAATCAACGTCGC) or replaced with a RhoA shRNA sequence (TGCTGTTGACAGTGAGCGCATTTCTTCCCACGTCTAGCTTAGTGAAGCCACAGATGTAAGCTAGACGTGGGAAGAAATTTGCCTACTGCCTCGGA). All virus stocks were propagated and titers were determined on NHDFs using standard techniques. For viral growth curves, NHDFs were infected at a multiplicity of infection (MOI) of 3 for single-step or an MOI of 0.01 for multistep for 2 h. Cell-associated and supernatant virus was harvested at multiple time points postinfection. Titers were determined by plaque assay on NHDFs.

### Reagents.

The 3′UTR of human RhoA was amplified by PCR from fibroblast genomic DNA using DNAzol and was cloned downstream of the *Renilla* luciferase gene in the psiCHECK-2 dual reporter construct (Promega) by XhoI and NotI restriction sites using the primer pair GCGGCTCGAGGTCTTGTGAAACCTTGCTGC and CGCCGCGGCCGCCTGCCTTTATTCTATTAGTAGTTGG. siGENOME RISC-free control siRNA (Neg; Dharmacon) and RhoA siRNA (s758; Thermo Fisher) were purchased for use in transfection experiments. Double-stranded miRNA mimics were custom designed and synthesized by Integrated DNA Technologies. The following commercial antibodies were used: α-mouse HRP, α-rabbit HRP, α-mouse IgG_1_-Alexa Fluor 488, GAPDH (ab8245; Abcam), HCMV IE2 (MAB810; Sigma-Aldrich), MLC (catalog no. 8505; Cell Signaling Technology), p-MLC (catalog no. 3671; Cell Signaling Technology), phalloidin-Alexa Fluor 647 (sc-363797; Santa Cruz Biotechnology), RhoA (ab54835; Abcam), and tubulin (200-301-880; Rockland).

### Luciferase assays.

HEK293T cells were seeded into 96-well plates and transfected with 100 ng of psiCHECK-2 vector and 100 fmol of negative control or miRNA mimic using Lipofectamine 2000 (Invitrogen). At 24 h after transfection, the cells were harvested for a luciferase assay using a Dual-Glo Reporter assay kit (Promega) according to the manufacturer’s instructions. Luminescence was detected using a Veritas microplate luminometer (Turner Biosystems). All experiments were performed in triplicate and are presented as means ± the standard deviations.

### Western blot analysis.

Cells were harvested in protein lysis buffer (50 mM Tris-HCl [pH 8.0], 150 mM NaCl, 1% NP-40, and protease inhibitors), loading buffer (4× Laemmli sample buffer with 2-mercaptoethanol) was added, and lysates were incubated at 95°C for 5 min. Extracts were loaded onto 4 to 15% acrylamide gels (Bio-Rad), transferred to Immobilon-P membranes (Millipore), and visualized with the specified antibodies. The relative intensity of bands detected by Western blotting was quantified using ImageJ software.

### Microscopy.

NHDFs or HEK293 cells were grown on 13 mm glass coverslips and transfected with pSIREN vectors or miRNA mimics using Lipofectamine 2000 or Lipofectamine RNAiMAX, respectively, according to the manufacturer’s instructions. Coverslips were washed with PBS and fixed with 4% paraformaldehyde in PBS. Cells were permeabilized with 0.2% saponin, blocked with BSA, and stained with the indicated primary antibodies. Coverslips were then washed with PBS containing BSA and 0.2% saponin, followed by incubation with the appropriate fluorophore-conjugated secondary antibodies. For some experiments, cells were treated with 1 μg/ml nocodazole for 20 h, followed by 0.1 μg/ml latrunculin A for 30 min, and then chased with fresh media for 0 to 8 h before fixation. Fluorescence was visualized using an EVOS FL autoimaging system using a 40× objective and 4′,6′-diamidino-2-phenylindole (DAPI), green fluorescent protein (GFP), and Texas Red filter cubes or a Leica SP5 scanning confocal microscope using the 63× objective with an NA of 1.4. The fluorophores were excited using 405, 488, 594, and 647 lasers. The signals were captured using Leica SP5 PMT in a sequential scan mode using the Leica Application Suite software. Images were exported as .tiff files and analyzed using ImageJ software.

### Proliferation assays.

NHDFs were transfected with 100 fmol of miRNA mimic, siRNA, or negative control using Lipofectamine RNAiMAX according to the manufacturer’s instructions. After 72 h, 5 × 10^3^ cells were plated in duplicate in 12-well plates. Proliferation was assessed at the indicated time points by trypan blue exclusion and manual counting.

### CD34^+^ HPC proliferation assays.

Freshly isolated or viably cryopreserved primary CD34^+^ HPCs were recovered overnight in stem cell media (Iscove modified Dulbecco medium [IMDM] containing 1% FBS, penicillin/streptomycin/glutamine [PSG], and stem cell cytokines [SCF, FLT3L, IL-3, and IL-6] [PeproTech]). HPCs were infected with HCMV at an MOI of 3 in IMDM containing 1% PSG, 10% BIT serum replacement (Stem Cell Technologies), stem cell cytokines, 50 μM β-mercaptoethanol, and 50 ng/ml low density lipoproteins (Calbiochem). All treated HPCs were isolated by fluorescence-activated cell sorting (BD FACSAria equipped with 488, 633, and 405 lasers, running BD FACSDiva software) for a pure population of viable, CD34^+^, GFP^+^ HPCs. Pure populations of sorted HPCs were plated at 10^4^ cells/ml in progenitor cell proliferation media (SFEMII; Stem Cell Technologies) supplemented with penicillin/streptomycin, 10% BIT, and stem cell cytokines at 200 μl/well in triplicate in 96-well plates for proliferation assays. Proliferation was assessed at 2, 5, and 7 days postplating (4, 7, and 9 dpi) by trypan blue exclusion and manual counting. For proliferation during latency, HCMV latency was established in long-term cultures of CD34^+^ HPCs using previously detailed methods (87). Briefly, isolated HPCs were cocultured in transwells above monolayers of irradiated M2-10B4 and S1/S1 stromal cells for 12 days. Proliferation was assessed at 12 days postplating (14 dpi) by trypan blue exclusion and manual counting.

### Quantitative PCR for viral genomes.

DNA from CD34^+^ HPCs was extracted using the two-step TRIzol (Thermo Fisher) method according to the manufacturer’s directions. Total DNA was analyzed in triplicate using TaqMan FastAdvanced PCR master mix (Applied Biosystems) and the primer and probe for HCMV *UL141* and human β-globin as previously described (92). The copy number was quantified using a standard curve generated from purified HCMV BAC DNA and human β-globin containing plasmid DNA, and data were normalized per cell assuming two copies of β-globin per cell.

### Statistical analysis.

Statistical analysis was performed using GraphPad Prism software (v6 or v7) for comparison between groups using a Student *t* test and one-way or two-way analysis of variance (ANOVA) with a Tukey’s *post hoc* test, as indicated. Values are expressed as means ± standard deviations or standard errors of the mean, as indicated in the figure legends. Significance was accepted with *P* < 0.05.
